# Does public pension crowd out the participation of older adults in community volunteering? Evidence from China

**DOI:** 10.3389/fpubh.2025.1533922

**Published:** 2025-03-25

**Authors:** Zhi-Yun Li, Ying-Yi Tang, Hua-Lei Yang, Li-Li Tang

**Affiliations:** ^1^College of Politics and Public Administration, Qingdao University, Qingdao, China; ^2^School of Public Administration, Zhongnan University of Economics and Law, Wuhan, China

**Keywords:** active aging, public pension, older adults, volunteering participation, crowding-out effects

## Abstract

**Background:**

To address aging and its associated social risks, the World Health Organization proposed an “active aging” policy framework in 2002, highlighting “health, participation, and security” as the three pillars for enhancing quality of life in old age. Extensive research has shown that public pensions, as a state-implemented social security measure, can effectively improve the health and well-being of older adults. However, existing studies have not sufficiently examined the causal impact of public pensions on social participation among older adults, such as community volunteering.

**Methods:**

Using data from the China Longitudinal Aging Social Survey in 2018, this article employs propensity score matching (PSM) and control function (CF) approaches to empirically examine the impact of the Urban and Rural Resident Social Pension (URRSP) on the participation of older adults in community volunteering. Specifically, we investigate whether this relationship varies across social groups.

**Results:**

Empirical results indicate that, compared to uninsured older adults, those enrolled in the pension program show a significantly lower likelihood and frequency of volunteering. This finding is robust after controlling for observable and unobservable characteristics, thus supporting the crowding-out hypothesis. Furthermore, heterogeneity analysis reveals that this crowding-out effect is more pronounced among older adults with higher socioeconomic status, such as those living in urban areas, with higher education, higher income, and better health. In other words, the limited benefits provided by the URRSP not only fail to offer financial support for volunteering, but also significantly reduce the willingness and level of volunteering among those with higher socioeconomic status.

**Conclusion:**

The above findings confirm the crowding-out hypothesis, suggesting that in developing countries with underdeveloped and stratified social security systems, state-led public pensions may crowd out older adults’ participation in volunteer activities that are altruism and mutual support.

## 1 Introduction

Population ageing and the associated social risks have become a significant governance challenge for many industrialized countries. In response to this challenge, the WHO proposed the “Active Ageing” policy framework, which highlights “health, participation, and security” as the three pillars for enhancing quality of life in old age (1). Within this framework, social participation of older adults is considered a core ingredient, aiming to encourage their involvement in social, cultural, and spiritual activities. The active aging model envisions older adults not only as beneficiaries of social services but also as active participants who can contribute to society through various forms of paid and unpaid work (2). Based on this initiative, many developing countries have implemented social security and welfare policies to address poverty and vulnerability in old age (3). Among these measures, public pensions serve as a fundamental form of security, providing essential economic support to older adults, thereby fostering a foundation for improved health and social participation. Extensive empirical research has shown that social pensions can effectively improve the health and well-being of older adults (4). However, existing studies have not fully examined the causal impact of public pensions on the social participation of older adults, such as their involvement in volunteer activities.

Conceptually, as an important form of social participation, community volunteering refers to productive and altruistic activities (5) in which older adults voluntarily engage in activities and services that benefit the local community (6). Volunteering among older adults is a crucial pathway to achieving active aging, as it not only brings health benefits to older adults (7) but also contributes positively to the community (8). Specifically, by offering help to those in need, older adults who participate in volunteer activities expand their social networks, gain increased social support, and, in turn, obtain a form of informal insurance against future risks^1^.

Theoretically, there are two competing views regarding the relationship between state-led pensions and community volunteering among older adults. The first is *the crowding-out hypothesis*, which suggests that welfare states and their generous social spending take responsibility away from civil society and individuals, thereby impeding the formation of social ties and social capital (9). According to this view, public pensions provide an alternative form of risk-sharing for older adults, thus weakening their motivation to engage in mutual support activities (7). Alternatively, *the crowding-in hypothesis* posits that public pensions not only offer economic security and income support for older adults to participate in volunteer activities but also narrow income gaps across social strata, fostering a culture of solidarity and mutual assistance. Consequently, state-led pensions are believed to increase the willingness of older adults to participate in volunteering (10).

Empirically, much of existing literature largely employs cross-national comparisons to examine the relationship between welfare policies and volunteering participation, yet the findings remain inconclusive. Some studies have found significant evidence of the crowding-in effect. For instance, Ackermann et al.’s analysis of 23 European countries found that volunteering is higher in more generous welfare states. In particular, pension welfare significantly strengthens volunteering among the retired and older adults (11). Similarly, Visser et al. found that higher social spending on healthcare and old-age pensions promotes social contact among older adults (12). On the other hand, other studies have provided evidence supporting the crowding-out view. For example, Stadelmann-Steffen found that the scale of welfare spending has a negative impact on volunteer participation, particularly for groups with higher socioeconomic status (9). Suzuki also observed that large-scale austerity measures by the government significantly raise the likelihood of individual volunteer participation, suggesting a substitution effect (13). Additionally, some studies have found no significant impact of welfare provision on civic volunteerism (9, 14). This inconsistency in findings underscores the need for further examination of these competing views.

However, existing researches have the following limitations. First, most studies focus on developed countries, with limited attention paid to developing countries. In developed countries, the formal welfare system, characterized by generous spending on pensions, healthcare, and social services, effectively meet the vast majority of citizens’ security needs (15). In contrast, in developing countries with less developed social protection systems, individuals rely more heavily on informal social support from relatives, friends, and community members to establish economic safety nets and sustain livelihoods, such as through altruistic donations and community volunteering. Consequently, the crowding-out effect of welfare policies is more likely to occur in developing countries. Second, scholars have argued that it is inappropriate to assume that welfare policies have uniform effects on all individuals (12, 16). In other words, the impact of welfare policies may vary across social groups. Third, considering the inability to control for all confounding variables that simultaneously affect both welfare provision and voluntary participation, previous studies using cross-national comparison methods may be biased. Additionally, estimates based on country-level data may suffer from ecological fallacy. Therefore, it is necessary to examine the causal effects at the micro level to complement the current research findings (10, 17).

As the largest developing country, China provides an ideal case for examining the relationship between pension programs and volunteer participation among older adults. On the one hand, to address poverty and improve quality of life, the Chinese government has established the world’s most extensive basic pension system. This system currently includes three types of basic pension programs targeting different populations: the Urban–Rural Resident Social Pension (URRSP), the Enterprise Employee Basic Pension (EEBP), and the Government and Institution Pension (GIP). This study focuses on the URRSP, which serves jobless urban and rural residents. Unlike employer-funded pensions, the URRSP is financed by subsidies from both central and local governments (18) and can be considered a poverty-alleviation policy with transfer-payment characteristics. In terms of pension benefits, China’s basic pension system exhibits a typical stratified structure, resembling a pyramid (19). This design not only causes “identity segmentation” but also exacerbates income inequality among older adults (20). As of 2022, the average benefit level for basic pensions was 1,745 CNY per month. However, the average monthly benefit for EEBP participants was 3,605 CNY, while for URRSP participants, it was just 204 CNY (21). On the other hand, given the low benefit levels of the urban–rural residents’ basic pension, which are insufficient to support basic livelihood (22), older individuals covered by this insurance still rely on informal social support from family, friends, and the community to cope with life risks (7, 23). Within the context of China’s stratified social insurance system, limited and unequal pension income fails to provide the necessary economic resources to support older adults’ participation in volunteer activities. Instead, it tends to amplify perceptions of social distance between individuals, undermines the foundation of trust, community norms, and collective consciousness, ultimately reducing individuals’ willingness to invest in social relationships with peers and neighbors (24). Consequently, we hypothesize that the URRSP, being at the bottom of the stratified pension system, will have a significant crowding-out effect on volunteer participation among older adults.

In summary, using the 2018 data from the China Longitudinal Aging Social Survey (CLASS), this article employs propensity score matching and the control function approach to empirically examine the impact of the Urban–Rural Resident Social Pension (URRSP) on community volunteering among older adults. In particular, we investigate whether this relationship varies across social groups. The paper is structured as follows: Section 2 introduces the data and methodology; Section 3 presents the empirical results and heterogeneity analysis; Section 4 provides interpretations and discussions of the findings; and Section 5 offers concluding remarks and policy implications.

## 2 Materials and methods

### 2.1 Data sources

The data used in this article was from the 2018 China Longitudinal Aging Social Survey, which was released by the Institute of Gerontology at Renmin University of China. By using a stratified multi-stage sampling method, the data were collected from respondents in 476 village (neighborhood) committees across 30 provincial administrative units within China. The survey content includes information on the health, family relationships, social background, and economic conditions of individuals aged 60 and above, with a particular focus on social participation among older adults, including volunteer services. A total of 11,419 respondents were collected in this survey. For the purposes of this study, we excluded older individuals who enrolled in the Enterprise Employee Basic Pension or the Government and Institution Pensions, and handled missing data. Ultimately, 7,606 valid samples were obtained. Data analysis was conducted using Stata 16.0.

### 2.2 Variables

#### 2.2.1 Dependent variable

The dependent variable is the community volunteering of older adults. In this survey, respondents were asked to recall whether they participated in a series of volunteer activities in the past year: “community security patrols, caring for other older people or children (such as helping with shopping, daily care, etc.), environmental sanitation and protection, mediation of neighborhood disputes, accompanying chats, volunteering services that require professional skills (such as free clinics, cultural and technological promotion, etc.), helping to look after other people’s children, and other activities?” For each activity, respondents chose the frequency form five options including “never,” “several times a year,” “at least once a month,” “at least once a week,” and “almost daily.” Further, we recode the above frequency variables into 0 (never), 1 (several times a year), 2 (at least once a month), 3 (at least once a week), and 4 (almost daily). Based on this, this study applies two volunteering indicators. The first indicator is a binary variable, namely *participation behavior*, which takes the value of 0 when the respondent had not undertaken given activities and takes the value of 1 when the respondent had participated in any given community activity. The second indicator is a continuous variable, namely *participation frequency*, which is measured by the sum of the participation frequencies of all activities. A higher score indicates a higher level of community volunteering by older adults.

#### 2.2.2 Independent variable

The core explanatory variable is whether older adults are beneficiaries of Urban and Rural Resident Social Pension (URRSP). Respondents were asked “Are you eligible to receive the basic pension from the URRSP?” If the answer is yes, then it takes the value 1, i.e., a *pensioner*, while if there is no any pension insurance, then it takes the value 0, i.e., a *non-pensioner*.

#### 2.2.3 Control variables

Following prior studies, we also control a series of confounding variables that may simultaneously affect older adults’ choice of participating in pension insurance and their community activities. Individual-level variables include gender, age (centered) and its square, place of residence (urban is 1, rural is 0), marital status (married with a spouse is 1, otherwise it is 0), education level (junior high school and above is 1, otherwise it is 0), personal total income last year (low-income group, middle-income group, high-income group and a category for missing values^2^), number of chronic diseases, subjective health level (1–5), employment status; family characteristics include the number of children, co-residence with children, number of grandchildren, etc. Descriptive statistics for all variables are shown in Table 1.

**Table 1:** Descriptive statistics.

	Non-pensioners (*N* = 2,837)	Pensioners (*N* = 4,769)	T-test
Variables	Mean\proportion	Mean\proportion	Difference
Participation behavior	0.417	0.281	0.135***
Participation frequency	3.367	2.275	1.092***
Female	49.30%	51.50%	−0.022*
Urban	75.10%	76.40%	−0.0130
Age	71.65	71.39	0.262
Married	68.20%	66.30%	0.036***
Education: middle school and above	21.40%	19.00%	0.024**
Low-income group	21.11%	19.35%	***
Middle-income group	28.45%	32.38%	
High-income group	22.52%	27.57%	
Income: Missing	27.92%	20.70%	
Number of chronic diseases	1.366	1.593	−0.228***
Employed	31.70%	34.10%	−0.024**
Health	3.300	3.277	0.0240
Number of grandchildren	3.165	3.191	−0.0260
Number of children	2.779	2.867	−0.088**
Live with children	32.60%	37.70%	−0.051***

*p < 0.1, **p < 0.05, ***p < 0.01.

### 2.3 Model

#### 2.3.1 Benchmark regression model

In the analysis, older adults’ volunteering participation is divided into participation behavior and participation frequency, which are binary and continuous variables, respectively. Therefore, the Probit model and OLS model are used as the baseline regression models to estimate the relationship between pension and participation in volunteer activity among older adults. The model can be constructed as shown in Equation 1.


(1) $$y_{i}=\beta_{0}+\beta_{1}\mathrm{pensio}n_{i}+\beta_{2}Z_{i}+\mu_{i}$$

where $y_{i}$ denotes whether the respondent *i* had participated in any volunteer activity over the past year or the sum of the participation frequencies of all volunteer activities. $\mathrm{pensio}n_{i}$ denotes whether the respondent *i* was enrolled in the Urban and Rural Resident Social Pension (URRSP), $Z_{i}$ represents a set of potential confounding variables, and $\mu_{i}$ is the random error term.

#### 2.3.2 Propensity score matching

Whether older adults participate in pension program is obviously a self-selection behavior, which depends on individual characteristics (observed and unobserved). In this case, the estimation results of the benchmark regression model could be biased. This study employs the propensity score matching method to test the robustness of the benchmark regression results, which can correct the selective bias caused by individual observed characteristics. The average treatment effect of participating in pension (ATT) can be obtained through Equation 2.


(2) $$ATT=\;[E(y_{1i}|D_{i}=1,p\left(Z_{i}\right)]-E[(y_{0i}\;|D_{i}=0,p\left(Z_{i}\right)]$$

Here, $y_{1i}$ represents the volunteering participation level of treatment group (*pensioners*), $y_{0i}$ represents the volunteering participation level of the control group (non-*pensioners*), $D_{i}=1$ denotes that older adults had participated in the URRSP, $D_{i}=0$ denotes that older adults had not participated in any pension insurance, and $p\left(Z_{i}\right)$ is the propensity score, which is estimated by the Logit model incorporating a series of observed variables.

#### 2.3.3 Control function method

The PSM method partially corrects the issue of endogeneity caused by observed variables; however, omitted variables and causality issues may still cause endogenous problems in the estimation (25). Based on Wooldridge’s recommendation (26), this study employs the control function (CF) approach using a two-stage estimation method to solve the endogeneity problem of pension participation. The CF approach involves predicting residuals from a first-stage regression model determining pension, and one or more valid instrumental variables are included (27). Inspired by the existing literature (28, 29), the individual’s choice of participation in URRSP is influenced by peer effects. Therefore, this study uses the take-up rate of URRSP in respondent’s community as an instrumental variable, denoted as $\mathrm{pension}\_comm_{i}$ . The first-stage estimation equation of the CF method can be expressed as in Equation 3.


(3) $$\mathrm{pensio}n_{i}=\alpha_{0}+\alpha_{1}\mathrm{pension}\_comm_{i}+\alpha_{2}Z_{i}+\varepsilon_{i}$$

In our analysis, since the pension is binary, the CF approach uses generalized residuals (GR) predicted from the Probit model. Then, we add the generalized residuals obtained from the first-stage regression model determining pension as additional covariate into the second-stage estimation model, as shown in the following equation:


(4) $$y_{i}=\beta_{0}+\beta_{1}\mathrm{pensio}n_{i}+\beta_{2}Z_{i}+\beta_{3}GR_{i}+\mu_{i}$$

One advantage of the CF approach is that it provides a simple, robust test of the null hypothesis that *pension* is exogenous. If the coefficient of *GR* in Equation 4 is statistically significant, indicating that *pension* is endogenous. In this case, the generalized residuals term needs to be included as a control variable in the volunteer participation model to correct for endogeneity bias. Conversely, if the coefficient of *GR* is not significant, the hypothesis of exogeneity of pension participation cannot be rejected, and then Equation 1 can achieve unbiased estimation.

## 3 Results

### 3.1 Descriptive analysis results

Table 1 shows the statistical analysis of all variables according to the treatment group and the control group (i.e., Pensioners vs. Non-pensioners). The mean test shows that, on the one hand, the proportion of older people participating in volunteer activities in the pensioners group is 28.1%, which is statistically significantly lower than the 41.7% of the non-pensioners group; on the other hand, the mean frequency of participation in volunteer activities of the pensioners group is 2.275, which is also statistically significantly lower than the 3.367 of the non-pensioners group. The robustness of these results requires further analysis. In addition, there are significant differences between the treatment group and the control group in most observable variables, which suggests that we need to correct possible endogenous problems such as selective bias.

### 3.2 Benchmark regression results

The above analysis shows that the volunteering participation of pensioners is significantly lower than that of the non-pensioners. Next, we add more control variables and uses Probit and OLS models to estimate the effects of pension insurance on the voluntary participation behavior and frequency of older adults. As shown in Table 2, when controlling for a series of covariates, participating in social pension significantly reduces the level of voluntary participation of older adults at the 1% level, which supports the crowding-out effect hypothesis.

**Table 2:** The impacts of pension on volunteering participation among older adults (probit & OLS).

	Participation behavior		Participation frequency	
	(1)	(2)	(3)	(4)
Pension receipt	−0.369*** (0.031)	−0.345*** (0.032)	−1.092*** (0.114)	−0.908*** (0.111)
Female		0.062* (0.032)		0.085 (0.108)
Urban		−0.007 (0.038)		−0.352** (0.138)
Age_c		0.003 (0.003)		0.044*** (0.011)
Age_c2		0.000 (0.000)		−0.000 (0.001)
Married		−0.021 (0.036)		−0.094 (0.121)
Education: Middle school and above		0.193*** (0.039)		0.497*** (0.140)
Low-income group		−0.265*** (0.047)		−0.914*** (0.123)
High-income group		0.231*** (0.042)		0.886*** (0.143)
Income: missing		0.301*** (0.042)		1.481*** (0.157)
Number of chronic diseases		−0.109*** (0.013)		−0.430*** (0.043)
Employed		−0.095*** (0.035)		−1.085*** (0.111)
Health		−0.004 (0.018)		−0.084 (0.063)
Number of grandchildren		0.069*** (0.016)		0.410*** (0.056)
Number of children		0.003 (0.015)		0.115** (0.055)
Live with children		−0.144*** (0.034)		−0.435*** (0.115)
Constant	−0.211*** (0.024)	−0.360*** (0.117)	3.367*** (0.094)	2.661*** (0.424)
Observations	7,606	7,606	7,606	7,606
R-squared	-	-	0.012	0.097

*p < 0.1, **p < 0.05, ***p < 0.01. The age variable has been centered. Robust standard errors are in parentheses.

In addition, in terms of control variables, variables such as being female, age, education level, income level, and number of grandchildren are positively correlated with the level of volunteering participation of older adults, while variables such as living in urban area, being employed, and living with children are negatively correlated with the participation of older adults in volunteer activities. These findings are consistent with existing research (30).

### 3.3 Robustness analysis

#### 3.3.1 PSM results

**Figure 1:**
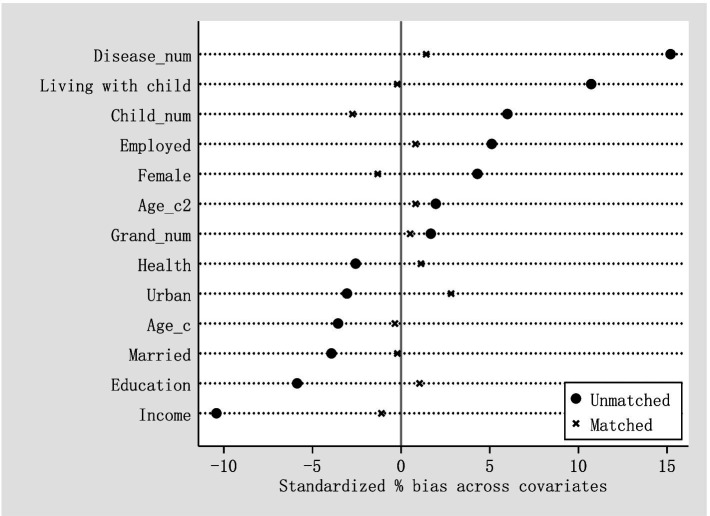
Standardized bias of each variable before and after matching.

As mentioned above, whether older adults participate in social pension is a self-selection behavior, so the above results may be a false correlation caused by selective bias. Therefore, we employ the propensity score matching method to estimate the average treatment effect (ATT) of the pensioners. The premise of the propensity score matching estimation is to meet the requirements of the covariate balance test (31). Figure 1 is a plot of the standardized bias of covariates before and after matching. After matching, the standardized bias of all covariates reduces significantly, and are all less than 5%, indicating that propensity score matching achieves high balance and similarity of covariates between the treated and control groups.

When the balance test conditions are met, the difference in community volunteer participation between the pensioners and the non-pensioners is the Average Treatment Effect on the Treated (ATT) as reflected by the treatment event. We employ different types of matching estimators to accurately identify the causal relationship between social pension and volunteering participation among older adults, including four-nearest-neighbor matching, caliper matching, and kernel matching (32). As shown in Table 3, the signs and significance of ATTs estimated by different matching method converge, indicating that the results are highly robust. Therefore, taking into account the selective bias caused by observable characteristics, participating in pension insurance still significantly reduces the likelihood and frequency of older people’s participation in voluntary activities.

**Table 3:** Regression results of PSM.

Matching methods	Participation behavior			Participation frequency		
	ATT	S.E.	Z	ATT	S.E.	Z
K-nearest neighbor matching	−0.127***	0.013	−9.990	−0.861***	0.128	−6.720
Radius matching	−0.128***	0.011	−11.160	−0.936***	0.116	−8.070
Kernel matching	−0.121***	0.012	−10.500	−0.860***	0.116	−7.390

*p < 0.1, **p < 0.05, ***p < 0.01. Bootstrap has a sample number of 300.

#### 3.3.2 CF results

The PSM method only solves the problem of selective bias caused by observable characteristics, but there may still be endogeneity problems caused by omitted variables or unobservable characteristics. Next, this study employs the control function method to estimate the impact of pension on the participation of older adults in volunteer activities. As shown in Table 4, firstly, the coefficient of the generalized residual (GR) is positive and statistically significant at the 1% level, which suggests that the endogenous issues of pension are well handled. Secondly, the coefficients of pension receipt on voluntary participation behaviors and frequency are-0.572 and-1.584, respectively, supporting the crowding-out effect hypothesis. In addition, the estimated coefficient based on the CF method is greater than that of the benchmark regression model, which shows that the OLS regression underestimates the negative impact of pension insurance on the participation of older adults in volunteer activities.

**Table 4:** The impacts of pension on volunteering (CF approach).

	Participation behavior	Participation frequency
	(1)	(2)
Pension receipt	−0.572*** (0.042)	−1.584*** (0.140)
Control variables	Yes	Yes
Generalized residual of first stage	0.275*** (0.036)	0.848*** (0.143)
Constant	−0.244** (0.119)	3.010*** (0.426)
Observations	7,606	7,606
R-squared	-	0.103

*p < 0.1, **p < 0.05, ***p < 0.01. Robust standard errors are in parentheses.

#### 3.3.3 Heterogeneity analysis

The above analysis indicates that the Urban–Rural Resident Social Pension has a significant crowding-out effect on the participation of older adults in voluntary activities. Next, based on the control function method, this study introduces interaction terms to examine the heterogeneous effects of URRSP on older adults’ volunteering according to region, education, health and income level. The results are shown in Table 5.

**Table 5:** Heterogeneity effects of the impacts of pension on volunteering (CF approach).

	Participation behavior				Participation frequency			
Variables	(1)	(2)	(3)	(4)	(5)	(6)	(7)	(8)
Pension receipt	−0.575*** (0.045)	−0.545*** (0.045)	−0.230* (0.121)	−0.653*** (0.063)	−1.429*** (0.145)	−1.407*** (0.150)	−0.501 (0.434)	−1.418*** (0.197)
Urban	−0.018 (0.058)				0.060 (0.226)			
Pension×Urban	0.015 (0.073)				−0.685** (0.267)			
Middle school and above		0.260*** (0.060)				0.999*** (0.233)		
Pension×Middle School		−0.128* (0.077)				−0.874*** (0.279)		
Health			0.060** (0.028)				0.128 (0.105)	
Pension×Health			−0.105*** (0.035)				−0.334*** (0.128)	
Low-income group				−0.498*** (0.075)				−1.538*** (0.205)
High-income group				0.301*** (0.070)				1.746*** (0.270)
Income: Missing				0.216*** (0.066)				1.729*** (0.253)
Pension×Low-income				0.370*** (0.095)				0.956*** (0.252)
Pension×High-income				−0.100 (0.086)				−1.275*** (0.309)
Pension×Missing				0.101 (0.085)				−0.560* (0.321)
Control variables	Yes	Yes	Yes	Yes	Yes	Yes	Yes	Yes
Generalized Residual of first stage	0.275*** (0.036)	0.276*** (0.036)	0.280*** (0.036)	0.291*** (0.036)	0.862*** (0.143)	0.850*** (0.142)	0.864*** (0.143)	0.958*** (0.144)
Observations	7,606	7,606	7,606	7,606	7,606	7,606	7,606	7,606
R-squared	-	-	-	-	0.104	0.105	0.104	0.109

*p < 0.1, **p < 0.05, ***p < 0.01. Middle income is the reference group. Robust standard errors are in parentheses.

The results in Table 5 again show that a crowding out tendency with older adults’ volunteering. More importantly, the results also demonstrate that public pension do not affect all individuals equally. From an urban–rural perspective, the results in Model (1) show that the interaction term between pension and urban residence is not significant, meaning there is no significant urban–rural difference in the likelihood of participation in volunteer activities. However, the results in Model (5) show that the interaction term coefficient is significantly negative, indicating that the negative impact of pension on volunteer participation frequency is significantly higher in urban areas than in rural areas. In other words, the crowding-out effect is significantly stronger among older adults living in urban areas compared to those living in rural areas.

From the perspective of educational differences, the results of Model (2) and Model (6) in Table 5 show that the coefficient of interaction term *Pension×Middle School* is significantly negative. This indicates that the adverse impact of pension on volunteering participation is significantly stronger among older individuals with higher educational attainment compared to those with lower educational levels.

From the perspective of health differences, the results of Model (3) in Table 5 indicate that the interaction term between pension and health status is negatively associated with volunteering. This suggests that the negative impact of pension on the likelihood of volunteering participation is significantly stronger among healthier older individuals compared to their less healthy counterparts. Furthermore, as shown in Model (7), while the main coefficient of pension is negative but not significant, the interaction term is significantly negative. This implies that the crowding-out effect of pension on volunteering frequency is primarily observed among older individuals with higher health capital.

From the perspective of income levels, the results of Model (4) and Model (8) in Table 5 show that the coefficient of interaction term *Pension×Low-income* is significantly positive. This indicates that, compared to the middle-income group, the crowding-out effect of pension on volunteering participation is weaker among older adults with low levels of income. Conversely, the interaction term *Pension×High-income* is positively associated with volunteering, suggesting a stronger crowding-out effect for older adults with high levels of income. Thus, the crowding-out effect of pension increases with rising income levels. It is important to note that for the low-income group, the coefficient for the effect of pension on volunteering is-0.473 (−1.418 + 0.945), which aligns with the crowding-out hypothesis. The above findings indicate that, within the context of China, the limited benefits provided by URRSP fail to generate an income effect sufficient to promote volunteering participation among all older individuals.

In conclusion, within China’s stratified social insurance system, the URRSP with the lowest benefit levels exerts a generally negative effect on community volunteering among older adults, supporting the crowding-out hypothesis. Notably, this crowding-out effect is more pronounced among older individuals with higher economic capital, human capital, and health capital.

## 4 Discussion

As the largest developing country, China has established the world’s largest social pension system in terms of coverage over the past few decades. However, this system exhibits distinct stratification, with significant disparities in benefits across different pension schemes, exacerbating income inequality among older adults. Among these, the Urban–Rural Resident Social Pension is at the bottom of the stratified pension system, covering the widest population but providing the lowest pension income, far below that of the other two types of public pension schemes (i.e., the GIP and the EEBP). In this context, this study focuses on the impact of the URRSP on volunteering participation among older adults and the heterogeneity of its effects.

Firstly, our study reveals that, overall, the Urban–Rural Resident Social Pension has a significant negative effect on the participation of older adults in community volunteering, supporting the crowding-out hypothesis. This can be attributed to several factors. On one hand, the benefits provided by the URRSP are limited and cannot cover the basic living standards, thus failing to exert the income effect. Under such circumstances, older adults still need to work to support themselves, lacking both financial resources and free time to engage in volunteer activities (24). On the other hand, the income inequality intensified by the stratified pension system undermines the foundation of trust, amplifies perceived cultural distance, and erodes the norms of mutual assistance. These factors collectively diminish the motivation of older individuals to participate in volunteering activities (20, 33). Additionally, as a poverty alleviation policy, the URRSP primarily targets populations without non-agricultural employment, which can lead to a welfare stigma effect. This diminishes beneficiaries’ perceived fairness, reducing their willingness to engage in volunteering (34).

Secondly, our study also finds that the crowding-out effect of the URRSP varies across different social groups. Specifically, the crowding-out effect on volunteer participation is significantly stronger among older individuals with higher socio-economic status (i.e., those with highly educated, healthy, and wealthy) compared to those with lower socio-economic status. Scholars have argued that we should not assume that welfare policies have the same impact on all groups (9). In general, participation in volunteer activities requires individuals to incur costs, while the benefits of volunteering are mainly immaterial, such as helping others and self-fulfillment (35). For higher social class, they not only have the economic resources and free time required to engage in volunteer activities, but they are also more motivated by the non-material rewards that such activities bring. As a result, they are more inclined to participate in volunteering (36). In liberal states with insufficient welfare support, privileged groups are motivated by altruism and moral obligation to help economically disadvantaged groups by offering private assistance and social support (37). However, when the state establishes basic pension systems and provides individuals with living security, this weakens the moral obligations and social responsibility of privileged groups, thus reducing their engagement in altruistic volunteer activities (17). Therefore, the establishment of social pensions has a stronger crowding-out effect on the volunteer participation of older adults in higher socio-economic groups. In contrast, for lower social classes, participation in social pension schemes can provide economic resources and reduce financial burdens, thereby enhancing their ability to engage in volunteer activities. Moreover, existing studies have shown that disadvantaged older individuals benefit more from community activities, such as improved health and lower risks of depression (4). As a result, the impact of social pension on the volunteer participation of lower socio-economic groups is not substantial, and may even have a positive effect.

Finally, due to data limitations, this study cannot identify the specific mechanisms through which social pension influence participation in volunteer activities. However, we can infer potential mechanisms by combining our findings with existing research. Most cross-national studies based on developed countries find that state welfare policies have a “crowding-in” effect on volunteering, and this effect is believed to be driven by two mechanisms: the resource mechanism and the cultural mechanism (11). Specifically, generous welfare policies not only provide economic resources and income security for volunteering, but they also foster a culture of mutual help, which in turn promotes citizen involvement in volunteer activities. The resource mechanism applies only to the beneficiaries of welfare policies, while the cultural mechanism affects all groups. Unlike findings from studies in developed countries, the crowding-out effect identified in this study suggests that the limited income provided by the URRSP fails to promote older adults’ volunteering participation through the resource mechanism. Furthermore, the universality of this crowding-out effect across all older individuals implies the operation of a cultural mechanism. Put differently, the stratified pension system and the income inequality it engenders undermine cultural norms of solidarity, equality, and justice that foster pro-social attitudes, which in turn suppress the participation of older adults in community volunteering (38).

## 5 Conclusion and policy implications

According to the strategic initiative of active aging, governments in developing countries worldwide are committed to establishing social security systems, especially pension system, to address the aging risks associated with industrial development. However, whether the establishment of social safety nets, as a formal welfare system, negatively impacts informal risk-sharing systems based on reciprocity—such as community volunteering—remains a subject of debate. This study uses data from the 2018 China Longitudinal Aging Social Survey and employs propensity score matching and control function methods to estimate the causal effect of the Urban–Rural Resident Social Pension on the participation of older adults in community volunteering. Our results show that, overall, participation in the URRSP has a significant negative effect on community volunteering among older adults, which supports the crowding-out hypothesis. This finding holds even after addressing issues such as selection bias and omitted variables. Furthermore, for older adults with higher socioeconomic status, the crowding-out effect of pension on volunteering would be stronger.

The findings of this study confirm the crowding-out hypothesis and provide some policy implications for promoting older adults’ volunteering to achieve active aging. Given that most studies suggest generous and universal welfare policies can enhance older adults’ social participation, including volunteer engagement (11), the Chinese government should increase fiscal subsidies to raise pension levels of the URRSP and reduce income disparities across different pension schemes. This approach would help promote community volunteering participation among older adults. Additionally, the government should allocate more resources to volunteer activities in urban and rural communities, establishing institutional safeguards and organizational support to encourage older adults’ active engagement in volunteering. Ultimately, these efforts would contribute to achieving the strategic goal of active aging.

However, this study has several limitations that warrant further research. First, as it relies on cross-sectional data from the 2018 CLASS survey, it cannot fully address the potential issue of reverse causality. Therefore, future research should employ longitudinal data to enhance the robustness of causality. Second, this study focuses solely on the URRSP and does not compare its impact with other pension schemes in China, such as the Enterprise Employee Basic Pension (EEBP) or the Government and Institution Pension (GIP). Examining the differential effects of various types of pension programs on older adults’ volunteering within a stratified social security system is a meaningful research question that requires further analysis. Finally, given the crucial role of cultural and social norms in shaping volunteering (39), future research should investigate the relationship between public pensions and older adults’ volunteering across different cultural and institutional contexts.

## Data Availability

Publicly available datasets were analyzed in this study. This data can be found here: http://class.ruc.edu.cn/index.htm.

## Footnotes

^1^However, the pension program offers an alternative form of risk sharing, which may weaken demand for social networking function of collective recreational activities and therefore lower realized participation in such activities. Second, altruistic activities involve providing social supports to those in need, partly in exchange for informal insurance against future risks.

^2^There are many missing values in the income variable, accounting for 23.4%. In order to preserve the sample information, this article retains the samples with missing income variables and divides them into a separate category.

## References

[1] World Health Organization. Active ageing: a policy framework. World Health Organization. (2002). Available online at: https://iris.who.int/handle/10665/67215 (Accessed March 15, 2024).

[2] Willie-Tyndale D Holder-Nevins D Mitchell-Fearon K James K Laws H Waldron NK. Participation in social activities and the association with socio-demographic and health-related factors among community-dwelling older adults in Jamaica. J Cross Cult Gerontol. (2016) 31:427–47. doi: 10.1007/s10823-016-9297-x, PMID: 27475790

[3] Willmore L. Universal pensions for developing countries. World Dev. (2007) 35:24–51. doi: 10.1016/j.worlddev.2006.09.008

[4] Chenxu N Shiyi G Yangyang P Zhen W. Sliver Age Action in the New Era: The Impact of Volunteer Service Participation on the Elderly’s Well-being. Chin J Popul Sci. (2023) 37:68–83.

[5] Morrow-Howell N Wang Y Amano T. Social participation and volunteering in later life. Oxford Res Encycl Psychol. (2019). doi: 10.1093/acrefore/9780190236557.013.404

[6] Sutan R Alavi K Sallahuddin SN Abdul Manaf MR Jaafar MH Shahar S. Factors associated with community volunteering among adults over the age of 50 in Malaysia. PLoS One. (2024) 19:1–14. doi: 10.1371/journal.pone.0302220 PMC11098473 38753828

[7] Meer T Scheepers P Grotenhuis M. States as molders of informal relations? A multilevel test on social participation in 20 Western countries. Eur Soc. (2009) 11:233–55. doi: 10.1080/14616690802133293

[8] Ågotnes G Moholt J-M Blix BH. From volunteer work to informal care by stealth: a ‘new voluntarism’ in social democratic health and welfare services for older adults. Ageing Soc. (2023) 43:2173–89. doi: 10.1017/S0144686X21001598

[9] Gundelach B Freitag M Stadelmann-Steffen I. Making or breaking informal volunteering. Eur Soc. (2010) 12:627–52. doi: 10.1080/14616696.2010.497224

[10] Gelissen J van Oorschot W Finsveen E. How does the welfare state influence individuals’ social capital? Eur Soc. (2012) 14:1–25. doi: 10.1080/14616696.2012.676660

[11] Ackermann K Erhardt J Freitag M. Crafting social integration? Welfare state and volunteering across social groups and policy areas in 23 European countries. KZfSS Kölner Zeitschrift Für Soziologie Und Sozialpsychologie. (2023) 75:283–304. doi: 10.1007/s11577-023-00881-8, PMID: 40082651

[12] Visser M Gesthuizen M Scheepers P. The crowding in hypothesis revisited: new insights into the impact of social protection expenditure on informal social capital. Eur Soc. (2018) 20:257–80. doi: 10.1080/14616696.2018.1442928

[13] Suzuki K. Government expenditure cuts and voluntary activities of citizens: the experience of Japanese municipalities. Asia Pacific J Public Adm. (2017) 39:258–75. doi: 10.1080/23276665.2017.1403179

[14] Van Oorschot W Arts W. The social Capital of European Welfare States: the crowding out hypothesis revisited. J Eur Soc Policy. (2005) 15:5–26. doi: 10.1177/0958928705049159

[15] Mumtaz Z. Comparing formal and informal social protection: exploring the usefulness of informal social protection in Pakistan. Inform Soc Prot Pov. (2022) 5:109–39. doi: 10.1007/978-981-19-6474-9_6

[16] Stadelmann-Steffen I. Social volunteering in welfare states: where crowding out should occur. Polit Stud. (2011) 59:135–55. doi: 10.1111/j.1467-9248.2010.00838.x

[17] Bradley E Chen X Tang G. Social security expansion and neighborhood cohesion: evidence from community-living older adults in China. J Econ Ageing. (2020) 15:100235. doi: 10.1016/j.jeoa.2019.100235, PMID: 34295643 PMC8294082

[18] Tao J. Can China’s new rural social pension insurance adequately protect the elderly in times of population ageing? Asian Public policy. (2017) 10:158–66. doi: 10.1080/17516234.2016.1167413

[19] Zhu H Walker A. Pension system reform in China: who gets what pensions? Soc Policy Adm. (2018) 52:1410–24. doi: 10.1111/spol.12368

[20] Li J Wang X Xu J Yuan C. The role of public pensions in income inequality among elderly households in China 1988–2013. China Econ Rev. (2020) 61:101422. doi: 10.1016/j.chieco.2020.101422

[21] Ministry of Human Resources and Social Security of the People’s Republic of China. Statistical Communiqué of the People’s republic of China on the 2022 human resources and social security. (2023) Available online at: http://www.mohrss.gov.cn/SYrlzyhshbzb/zwgk/szrs/tjgb/ (Accessed March 16, 2024)

[22] Shu L. The effect of the new rural social pension insurance program on the retirement and labor supply decision in China. J Econ Ageing. (2018) 12:135–50. doi: 10.1016/j.jeoa.2018.03.007

[23] Wood G Gough I. A comparative welfare regime approach to global social policy. World Dev. (2006) 34:1696–712. doi: 10.1016/j.worlddev.2006.02.001

[24] Ellwardt L Peter S Präg P Steverink N. Social contacts of older people in 27 European countries: the role of welfare spending and economic inequality. Eur Sociol Rev. (2014) 30:413–30. doi: 10.1093/esr/jcu046

[25] Pan G Li S Geng Z Zhan K. Do social pension schemes promote the mental health of rural middle-aged and old residents? Evidence from China. Front Public Health. (2021) 9:710128. doi: 10.3389/fpubh.2021.710128, PMID: 34395373 PMC8358066

[26] Wooldridge JM. Control function methods in applied econometrics. J Hum Resour. (2015) 50:420–45. doi: 10.3368/jhr.50.2.420

[27] Li H Li H Cao A Guo L. Does attending in social pension program promotes household energy transition? Evidence from ethnical minority regions of rural China. Energy Sustain Dev. (2022) 70:361–70. doi: 10.1016/j.esd.2022.08.013

[28] Chuanchuan Z Hanyu Z. The influence of the peer effect on participation in China's new rural pension scheme. J Financ Res. (2021) 9:111–30. (in Chinese)

[29] Zhao C Xi Q. Peer effects in pension decision-making: evidence from China’s new rural pension scheme. Labour Econ. (2021) 69:101978. doi: 10.1016/j.labeco.2021.101978

[30] Lee S. The volunteer and charity work of European older adults: findings from SHARE. J Nonprofit Publ Sect Market. (2024) 36:22–36. doi: 10.1080/10495142.2022.2130498

[31] Yang D Ren Z Zheng G. The impact of pension insurance types on the health of older adults in China: a study based on the 2018 CHARLS data. Front Public Health. (2023) 11:1180024. doi: 10.3389/fpubh.2023.1180024, PMID: 37333531 PMC10272461

[32] Abadie A Drukker D Herr JL Imbens GW. Implementing matching estimators for average treatment effects in Stata. Stata. (2004) 4:290–311. doi: 10.1177/1536867X0400400307

[33] Burchi F Loewe M Malerba D Leininger J. Disentangling the relationship between social protection and social cohesion: introduction to the special issue. Eur J Dev Res. (2022) 34:1195–215. doi: 10.1057/s41287-022-00532-2, PMID: 35527722 PMC9059109

[34] Hessel P Bermeo López LM López Franco LC Ham A Pinilla-Roncancio M González-Uribe C. Association between social pensions with depression, social, and health behaviors among poor older individuals in Colombia. J Gerontol. (2021) 76:968–73. doi: 10.1093/geronb/gbaa195, PMID: 33165527

[35] Wilson J. Volunteering. Annu Rev Sociol. (2000) 26:215–40. doi: 10.1146/annurev.soc.26.1.215

[36] Brady HE Sidney V Schlozman KL. Beyond SES: a resource model of political participation. Am Polit Sci Rev. (1995) 89:271–94. doi: 10.2307/2082425

[37] Scheepers P Grotenhuis MT. Who cares for the poor in Europe? Micro and macro determinants for alleviating poverty in 15 European countries. Eur Sociol Rev. (2005) 21:453–65. doi: 10.1093/esr/jci032

[38] Reeskens T van Oorschot W. European feelings of deprivation amidst the financial crisis: effects of welfare state effort and informal social relations. Acta Sociologica. (2014) 57:191–206. doi: 10.1177/0001699313504231

[39] Lu P Xu C Shelley M. A state-of-the-art review of the socio-ecological correlates of volunteerism among older adults. Ageing Soc. (2021) 41:1833–57. doi: 10.1017/S0144686X20000082

